# Sperm whale acoustic abundance and dive behaviour in the western North Atlantic

**DOI:** 10.1038/s41598-022-20868-3

**Published:** 2022-10-07

**Authors:** Annabel Westell, Taiki Sakai, Robert Valtierra, Sofie M. Van Parijs, Danielle Cholewiak, Annamaria DeAngelis

**Affiliations:** 1 Under Contract to the Northeast Fisheries Science Center, National Marine Fisheries Service, National Oceanic and Atmospheric Administration, 166 Water Street, Woods Hole, MA 02543 USA; 2 Environmental Assessment Services, LLC, 350 Hills St., Suite 112, Richland, WA 99354 USA; 3 Under Contract to the Southwest Fisheries Science Center, National Marine Fisheries Service, National Oceanic and Atmospheric Administration, 8901 La Jolla Shores Drive, La Jolla, CA 92037 USA; 4 Marine Acoustics Inc., 2 Corporate Pl #105, Middletown, RI 02842 USA; 5 Northeast Fisheries Science Center, National Marine Fisheries Service, National Oceanic and Atmospheric Administration, 166 Water Street, Woods Hole, MA 02543 USA

**Keywords:** Conservation biology, Ecology

## Abstract

Sperm whales are an ideal species to study using passive acoustic technology because they spend the majority of their time underwater and produce echolocation clicks almost continuously while foraging. Passive acoustic line transect data collected between June and August 2016 were used to estimate a depth-corrected acoustic abundance and study the dive behaviour of foraging sperm whales in the western North Atlantic Ocean. 2D localizations (n = 699) were truncated at a slant range of 6500 m and combined with the multipath arrivals of surface reflected echoes to calculate 3D localizations (n = 274). Distance sampling using depth-corrected perpendicular distances resulted in a 10.5% change in the acoustic abundance estimate (2199 whales, CV = 14.6%) compared to uncorrected slant ranges (1969 whales, CV = 14.1%), and a detection function that was a better fit for the data. Sperm whales exhibited multiple foraging strategies, with bottom phases occurring at depths of 400–800, 800–1200, or > 1200 m, accounting for an average 39.2, 49.5, or 44.9% of the total recorded dive time, respectively. These results suggest that estimating 3D localizations using acoustic line transect data improves acoustic abundance estimation and can be used to answer population level questions about foraging ecology.

## Introduction

Sperm whales (*Physeter macrocephalus*) were hunted close to extinction in the whaling era and are currently classified as Endangered on the US Endangered Species List^1^ and Vulnerable on the IUCN Red List^2^. Understanding regional trends in sperm whale abundance, distribution, and foraging ecology is critical to develop effective management for and evaluating the recovery of this species^3^. Previous sperm whale abundance estimates in the western North Atlantic have primarily relied on visual surveys^4,5^, however these deep diving cetaceans are difficult to detect and count visually as they can spend more than 70% of their time in foraging dive cycles^6^. During a dive cycle a sperm whale can be submerged for more than an hour and spend only 8–10 min at the surface between dives^6–8^.

Fortunately, sperm whales are very acoustically active while underwater, producing loud, distinctive clicks classified based on the inter-click-interval (ICI) as usual clicks (ICI 0.2–2.0 s), buzzes (ICI < 0.2 s), codas (patterned social clicks), and slow clicks (ICI > 2.0–8.0 s)^9–12^. Usual clicks are produced in long trains and are used for echolocation during every foraging dive^13,14^. These clicks can be detected acoustically from 10 to 16 km away due to high source levels and a centroid frequency between 5 and 15 kHz^15,16^. As such, the occurrence of these clicks can be used to identify individuals and estimate sperm whale abundance via distance sampling.

Acoustic line transect surveys can be used to estimate sperm whale acoustic density and abundance^3,17,18^. Target motion analysis (TMA) uses the intersecting bearings to clicks to estimate the two-dimensional (2D) slant range between the detected whale and the trackline^19–21^. As most sperm whales are detected at depth, this method ignores the vertical component of their location. A TMA derived slant range will be greater than the true horizontal perpendicular distance at the sea surface and will be more biased as detections get closer to the trackline. This bias is commonly observed in acoustic studies of sperm whales^22,23^ but the assumption that it has a minimal impact on acoustic density estimation^19,24^ may not be true for every dataset, population, or habitat.

Sperm whales feed on a variety of meso- and bathypelagic fish and squid^25–27^ and are known to adapt foraging strategies depending on the habitat and prey type^28,29^. Animal tagging has resulted in an increased understanding of how sperm whales navigate their environment, including which layers of the water column are used for foraging and fine-scale predator–prey interactions^7,27–30^. Although they provide invaluable insights, tagging studies are often restricted to small sample sizes. Alternatively, 3D localization using passive acoustic towed array data yields information from a larger proportion of the population and thus can be used to draw broader conclusions about dive behaviour and foraging ecology. DeAngelis et al.^31^ combined 2D TMA localizations with the time difference between the arrival of a direct click and corresponding surface reflected echo on a towed hydrophone element to estimate the depth of echolocating beaked whales. As sperm whales also echolocate at depths greater than most towed hydrophone array depths, this analysis applied the same method to their usual clicks.

In this study, passive acoustic line transect data collected during the summer of 2016 as part of the Atlantic Marine Assessment Program for Protected Species (AMAPPS)^32^ were used to detect and localize sperm whales in 3D, compare uncorrected and depth-corrected acoustic abundance estimates, and study the foraging behaviour of the detected individuals. An analysis conducted by Cholewiak et al.^33^ found that during similar surveys the scientific echosounders had an effect on the detection of beaked whales. Therefore, a similar analysis was conducted to determine whether there was any effect on the acoustic detection of sperm whale usual click trains.

## Methods

### Data collection

Between June 27 and August 25, 2016, 6600 km of simultaneous visual and passive acoustic line transect surveys were completed on the National Oceanic and Atmospheric Administration (NOAA) ship *Henry B. Bigelow*^5^. Survey effort was distributed along saw tooth track lines spanning the continental slope from Virginia (US) to the southern tip of Nova Scotia (Canada) (36–42 N) and on several larger track lines over the abyssal plain. Two teams of visual observers independently recorded sightings of marine mammals using high-powered Fujinon binoculars (25 × 150; Fujifilm, Valhalla, NY) as well as environmental conditions (e.g. sea state) every 30 min.

The speed of sound in water was collected three times each day (morning, noon, evening) by measuring conductivity, temperature, and depth (CTD) at specific intervals in the water column. The sound speed closest to the depth of the towed hydrophone array was extracted. On alternating survey days, Simrad EK60 single beam scientific echosounders operating at frequencies of 18, 38, 70, 120 and 200 kHz were used to collect active acoustic data.

**Figure 1:**
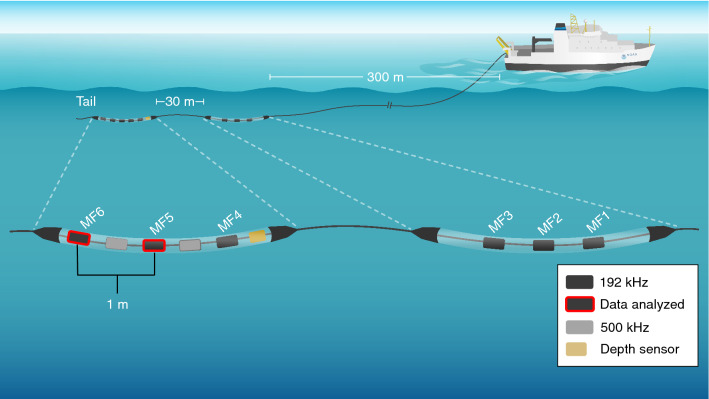
The linear towed array included eight hydrophone elements and a depth sensor within two oil-filled modular sections separated by 30 m of cable. Six hydrophones sampled at 192 kHz (MF1–MF6) and two sampled at 500 kHz. The hydrophones were connected to two National Instruments sound cards (NI-USB-6356). A high pass filter of 1 kHz was applied by the recording system to reduce the amount of vessel noise in the recordings. This analysis used the passive acoustic data from MF5 and MF6. The schematic is not to scale.

When possible during daylight hours (06:00–18:00 ET), passive acoustic data were collected continuously using a custom-built linear array composed of eight hydrophone elements and a depth sensor (Keller America Inc. PA7FLE, Newport News, VA) within two oil-filled modular sections separated by 30 m of cable (Fig. 1). The array was towed 300 m behind the vessel at approximately 5–10 m depth while the vessel was in waters more than 100 m deep and underway at speeds of 16–20 km/h. For more details see DeAngelis et al.^31^, with the only change being that two APC hydrophones and one Reson hydrophone in the aft section were replaced with HTI-96-Min hydrophones (High Tech, Inc., Long Beach, MS). The HTI’s had a flat frequency response from 1 to 30 kHz (− 167 dB re V/uPa ± 1.5 dB). Recordings were made using the acoustical software PAMGuard (v.1.15.02)^34^. This analysis used the data recorded by the last two 192 kHz sampled hydrophones in the array (MF5 and MF6).

### Click detection and 2D event localization

The passive acoustic data were filtered using a Butterworth band pass filter (4th order) between 2 and 20 kHz and decimated to 96 kHz to improve sperm whale click resolution. Clicks were automatically detected using the PAMGuard (v.2.01.03) general sperm whale click detector with a trigger threshold of 12 dB.

Using PAMGuard’s bearing time display, all detections were reviewed to classify click types and mark click trains as “events” based on consistent changes in bearing, audible sound, ICI and spectral characteristics. Each event was marked to an individual level, tracking a whale from the first to the last detected click^15,35^. All events containing usual clicks were localized with PAMGuard’s Target Motion Analysis (TMA) module’s 2D simplex optimization algorithm. For further analysis, events were truncated at a slant range of 6500 m (Supplementary Fig. S1).

### Echosounder analysis

A regression analysis was run using the R package MASS^36^. To account for overdispersion, a negative binomial generalized linear model (GLM) with a log link function was applied to a dataset of the daily acoustic detections^33^. Echosounder state (active versus passive), month (June, July, August), and habitat type (slope or abyssal) were included as covariates, with the total number of daily detections as the response variable. The track line distance covered per day was used as an offset for effort. The best fitting model was selected based on backwards stepwise selection using Akaike’s information criterion (AIC) and the single-term deletion method using Chi-squared goodness-of-fit tests^37^.

### 3D localization

#### Extracting a .wav clip for each click and attributing metadata

An automated process was developed using the R package PAMpal^38^ (v. 0.14.0) to extract the time of each click in the marked events from PAMGuard databases, generate a .wav clip for each click, and attribute all metadata (e.g., event 2D localization, array depth, radial distance, sea state, and sound speed) necessary for estimating the click depth.

#### Slant delay

**Figure 2:**
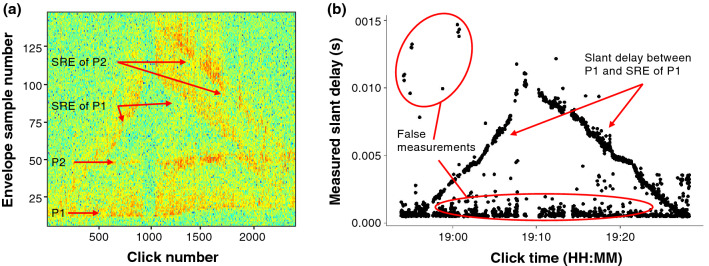
(**a**) Echogram illustrating the energy recorded in the 0.015 s after each of 2425 clicks in an event. The vertical axis includes 144 samples at a decimated frequency of 9600 Hz (0.015 s). The P1 and P2 pulses of the direct click are visible as are the surface reflected echoes (SRE) of these pulses. (**b**) Plot of the measured slant delay (s) over the click time (HH:MM). Slant delay increases to ~ 0.01 s and then decreases over a time period of approximately 34.5 min. Some of the false measurements of slant delay, not consistent with the pattern, are marked.

Using the methods established by DeAngelis et al.^31^ and custom Matlab R2021a (MathWorks Inc., Natick, NA) scripts, the multipath arrival of clicks and surface reflected echoes were used to mathematically convert the linear array into a 2D planar array and estimate 3D localizations. Using the .wav clips exported from PAMPal, the time delay between the click and the corresponding surface reflected echo, known as the slant delay, was measured via autocorrelation. Within the autocorrelation solution’s envelope of correlation values, the optimal slant delay was measured using the peak with the highest correlation value above a threshold of 0.02 and within an expected time window after the direct click of 0.0005–0.015 s. Although theoretically a surface reflected echo could have arrived less than a millisecond (< 0.00006 s) after a sperm whale’s click, a minimum time of 0.0005 s was applied because otherwise the autocorrelation solution consistently selected a peak within the direct click. In addition, as a result of the multi-pulse structure of sperm whale clicks^11,15^, the autocorrelation solution can select a peak from a second or third pulse of the direct click if it has a larger amplitude than the peak of the surface reflected echo. The inter-pulse interval (IPI) of a direct click can be consistent over short periods of time^39^, while the time between the direct click and surface echo can vary as the whale moves up or down in the water column. These patterns can occasionally be visualized and distinguished using an echogram (Fig. 2). Therefore, for each event a plot of the measured slant delay over event time was manually reviewed for expected patterns, and probable false detections were removed.

#### Average depth and standard deviation

For each click with a measured slant delay a click depth was estimated by combining the slant delay with the required metadata^31^. Click depth values were truncated at 4000 m as any greater measurements were likely based on false detections of the surface reflected echo. For each event, the click depths were used to calculate an average depth. The 3D localization was rejected if the average depth exceeded the seafloor depth^40^ where the event was recorded. A standard deviation was calculated based on the combined uncertainties from the slant delay, 2D localization, and depth measurement while adjusting for the number of clicks in an event.

#### Dive depth patterns

**Figure 3:**
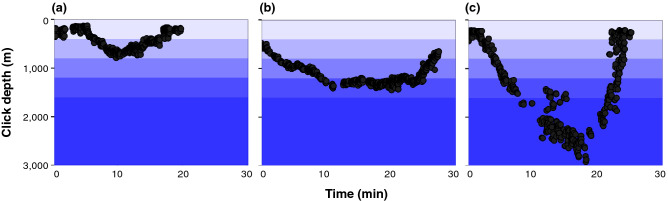
Example of click depths (m) over time (min) for events categorized as (**a**) U shaped and shallow (< 800 m), (**b**) U shaped and medium (800–1600 m), (**c**) U shaped and deep (> 1600 m).

For each event with a 3D localization, patterns in the click depths over time were manually reviewed and categorized. If a descent, bottom phase and ascent were present in the click depths (Supplementary Fig. S2), longer events (> 5 min) were categorized as U shaped, and as shallow (< 800 m), medium (800–1600 m), or deep (> 1600 m) based on the maximum click depth (Fig. 3).

Click depths were then binned at 400 m intervals to account for an animal’s unknown horizontal movement over time as well as uncertainty in the estimated click depths, and the total time an animal spent within each depth bin was calculated. For each event with a U shaped click depth pattern, the depth bin in which the bottom phase occurred^6^ was determined. Finally, to assess if a whale was diving in the water column or close to the seafloor, the depth bin in which the 90th percentile of the click depths was recorded was compared to the bin including the seafloor depth. If the whale was more than 400 m above the seafloor, it was determined to be diving in the water column.

### Distance sampling

#### Depth-corrected average horizontal perpendicular distances

For each event, a depth-corrected average horizontal perpendicular distance was calculated using the TMA derived perpendicular slant range and the average depth or an assumed depth in the Pythagorean theorem^19,31^. The weighted mean, first quartile, and third quartile of the average depths were tested as assumed depths for events excluded from 3D localization. If depth was greater than or equal to the slant range the perpendicular distance was coerced to 0, indicating the whale was diving directly below the track line. The resulting distribution of depth-corrected perpendicular distances that aligned most with distance sampling theory was used in the final distance analysis.

#### Acoustic density and abundance estimation

The R package Distance^41,42^ (v.1.0.4) was used to estimate two separate detection functions based on the uncorrected slant ranges and the depth-corrected perpendicular distances. Half-normal, uniform, and hazard rate key functions were tested with cosine, simple polynomial, and Hermite polynomial adjustment terms. The best fitting models were selected based on the AIC, the Kolmogorov–Smirnov (K–S) test, the Cramer-von Mises (CvM) test, quantile–quantile plots, and visual review of the fitted models^43^. The probability of detection, abundance, and effective strip half width (ESW) were then estimated for foraging sperm whales.

#### Permitting authority

Data used in this manuscript were collected during surveys that were completed under U.S. Marine Mammal Protection Act permit numbers 17355 and 21371 issued to the Northeast Fisheries Science Center.

## Results

Over 39 survey days a total of 354 h of on-effort acoustic recordings were collected along 5661 km of trackline. 3178 km (56.1%) of effort was completed over the continental slope (depth ≤ 2000 m) and 2483 km (43.9%) was completed offshore (depth > 2000 m). The daily average sound speed ranged from 1499 to 1543 m/s.

### Detections

**Figure 4:**
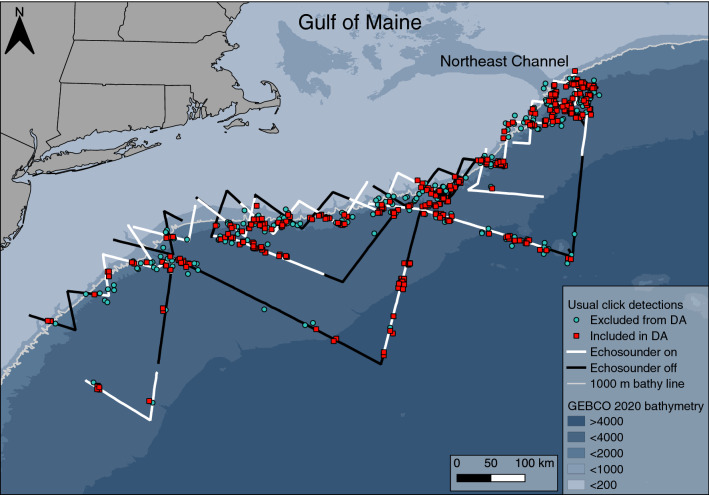
Map of the survey conducted between June 27 and August 25, 2016, in the western North Atlantic and covering 5661 km. Marked events are of sperm whale usual click trains (n = 712) coloured based on whether it was included (n = 431) or excluded (n = 281) from the distance analysis (DA) if 2D localization was not possible or after truncating at a slant range of 6500 m.

In total 768 events of sperm whales were classified, and 712 of these contained usual clicks (Fig. 4). Detections of click trains including only coda (45 events) or slow clicks (11 events) were not concentrated around a specific area or date and were not included in this analysis. Sperm whale events occurred every day and on 49 of the 54 transect lines, accounting for 95% of the survey effort. The highest number of usual click train events per kilometer were recorded on transects situated at the mouth of the Northeast Channel into the Gulf of Maine between July 24th and 26th. Clicks from an individual sperm whale were tracked and marked continuously for periods of < 1–68.6 min (mean = 10.7 min), with the number of clicks detected ranging from 7 to 4628 (mean = 593 clicks).

### Echosounder analysis

690 usual click events recorded over 38 survey days were included in the echosounder analysis (Table 1). The regression analysis revealed that echosounder state was not a significant factor in the number of acoustic detections or number of events suitable for 3D localization per day at the 95% significance level. Only month remained when using a backwards stepwise model selection process (Table 2) or was significant (*P* = 0.01286) using a single term deletion method. In addition, the mean number of events per day, hour, or kilometer surveyed did not vary significantly at the 95% level when the echosounder was active versus passive (*P* day = 0.578; *P* hour = 0.693; *P* kilometer = 0.759).

**Table 1:** Passive acoustic effort and acoustic detections of usual click trains when the echosounder was in active and passive modes.

	Echosounder active	Echosounder passive	Total
Number of days	18	20	38
Number of detections	295 (42.8%)	395 (57.2%)	690
Time analyzed (h)	159.9 (46.3%)	185.6 (53.7%)	345.5
Distance surveyed (km)	2931.5 (45.3%)	3534.3 (54.7%)	6465.8
Mean detections per hour	1.81	2.07	
Mean detections per km	0.098	0.112	

38 of the 39 survey days were included; August 24 was excluded because the echosounder state changed during the day.

**Table 2:** Stepwise AIC selection process to assess the relationship between echosounder status and number of acoustic detections of usual click trains.

	Resid. d.f	Resid. deviance	AIC	ΔAIC
Month	35	40.06	290.19	0.0*
Echosounder + month	34	39.98	291.58	1.39
Echosounder + month + habitat type	33	39.95	293.42	1.84

An asterisk indicates the best fit model. The starting model (full model) was count ~ echosounder + month + habitat type, offset by the log of the trackline distance to account for differences in effort.

### 2D localization

Perpendicular slant ranges between the trackline and localized sperm whales varied from 31 m (error = 6.7 m) to > 30 km (699 events) (Table 3). 2D localization was not possible for 13 (1.8%) events and rejected for 109 (15.3%) others based on a lack of convergence in the bearings. These click trains were often faint and intermittent, which could be a result of an animal being at the edge of the acoustic detection range, background noise, or changes in an animal’s sound output level, orientation, or position in the water column^44^. Truncating the remaining events at a range of 6500 m resulted in 431 events for distance sampling and depth estimation.

**Table 3:** Summary of the number of events containing sperm whale usual clicks included in each step of the analysis from click train detection to 3D localization.

	Number of events
Detected and marked	712
Localized in 2D using TMA	699
Passed manual review of 2D convergence	590
Slant range ≤ 6500 m	431*
Passed manual review of slant delay	274**

A single asterisk indicates the number of events used in distance analysis and double asterisks indicates the number of events included in 3D localization and dive depth calculation.

### 3D localization

#### Average dive depths

**Figure 5:**
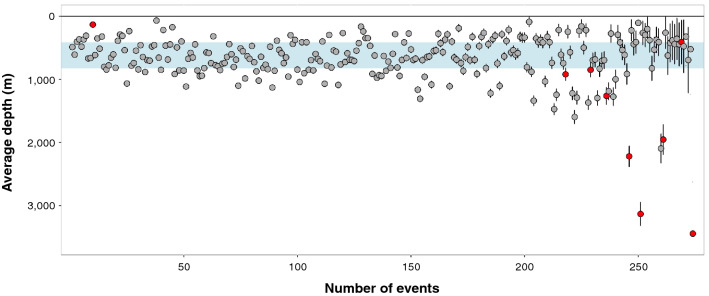
Average dive depth with depth error calculated for 274 events. The events are ordered based on the depth error value. The average depth was rejected for nine events (red points). The interquartile range for the entire dataset is highlighted in blue (first quartile = 411 m, third quartile = 821 m).

Slant delay was measured and manually reviewed for 431 events, and an average dive depth was calculated for 274 (63.6%) of these events (Fig. 5). The 3D localization was rejected for nine events because the average depth was greater than the seafloor depth. The number of clicks per event with a measured slant delay ranged from 8 to 1734 (mean = 527 clicks). The minimum and maximum average event depths were 70 m (SD = 24 m) and 2094 m (SD = 321 m). The weighted mean of all the event average depths was 620 m (SD = 280 m), and the first and third quartiles were at 411 m and 821 m.

#### Dive depth patterns

**Figure 6:**
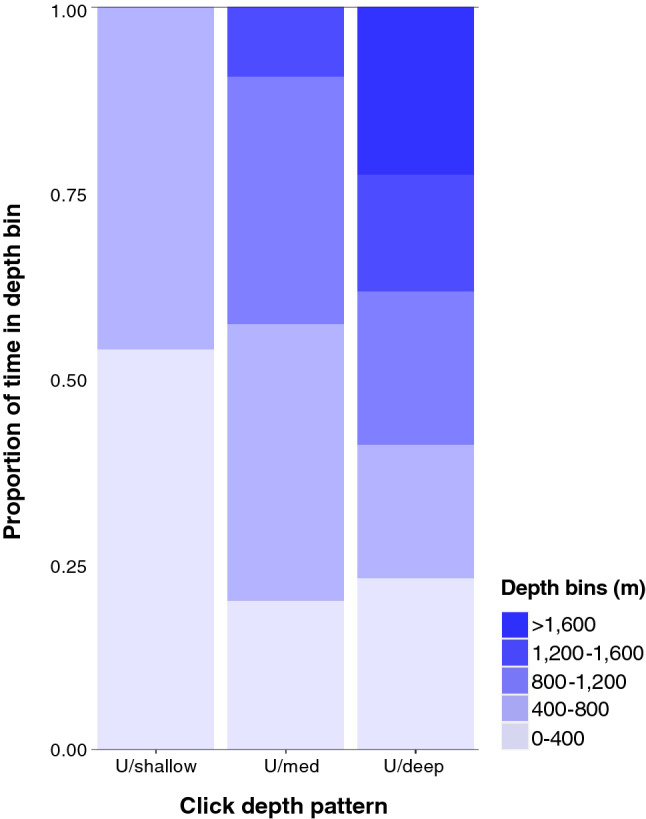
Proportion of time recorded in 400 m depth bins or at depths > 1600 m for events categorized based on patterns in click depths U shaped and shallow (n = 10), U shaped and medium (n = 25), or U shaped and deep (n = 9).

Of the 265 events with accepted 3D localizations, 44 (16.6%) were categorized as U shaped, and further divided based on maximum click depths as shallow (n = 10), medium (n = 25), or deep (n = 9) (Supplementary Table S1). The remaining events only included partial dives or lacked a clear pattern in click depths. These were labelled as flat and shallow (n = 9), ascending (n = 31), descending (n = 70), or no pattern/uncertain (n = 111). Bottom phases were noted at 400–800 m (n = 15 events), 800–1200 m (n = 18 events), and at depths > 1200 m (n = 10 events). On average, whales spent 39.2, 49.5, and 44.9% of the recorded time, respectively, in these sections of the water column. For the U shaped and shallow dives, almost equal time was recorded in the 0–400 m (53.9%) and 400–800 m (46.1%) depth bins (Fig. 6). For U shaped and medium depth dives, the majority of time was recorded in the 400–800 m (37.2%) and 800–1200 m (33.3%) depth bins. For U shaped and deep dives, similar proportions of time were recorded in each depth bin.

**Figure 7:**
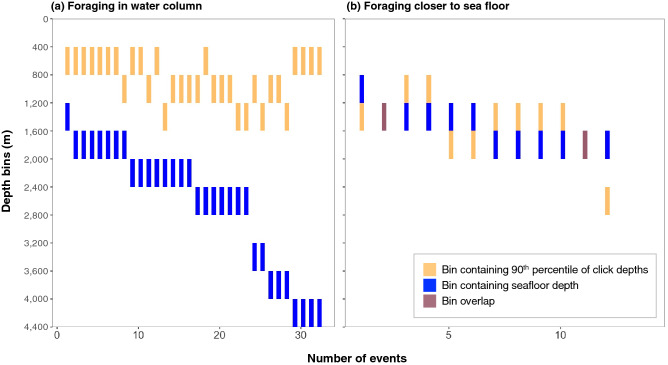
For the 44 events with a U shaped click depth pattern, the whale was categorized as diving in the water column if (**a**) the bin including the 90th percentile of the click depths was more than 400 m above the bin including the seafloor depth (n = 32 events) or was categorized as diving closer to the seafloor if (**b**) within 400 m of the bin including the seafloor depth (n = 12 events).

For 32 of the events (73%) with a U shaped click depth pattern, the whale was more than 400 m above the seafloor, indicating it was foraging in the water column (Fig. 7). For the remaining 12 events (27%) the whale was within 400 m of the seafloor. In four cases the bin including the 90th percentile of the click depths exceeded the bin including the depth of the seafloor, indicating the seafloor depth or the measurement of the deepest click depths were inaccurate.

### Distance analysis

#### Depth-corrected average horizontal perpendicular distances

The first quartile of the weighted average depths (411 m) was used as an assumed depth for nine events with average depth estimates greater than the seafloor, and for 157 events not included in 3D localization due to short duration (n = 28), lack of multipath surface reflected echoes (n = 128), or estimated click depths > 4000 m (n = 1). The depth-corrected perpendicular distance was coerced to 0 m for 34 events (7.9%). For most of the remaining events (92.7%), the depth-corrected perpendicular distance resulted in a decrease in perpendicular distance by < 20% compared to the corresponding uncorrected slant range. The weighted mean depth (620 m) and third quartile (821 m) were tested as assumed depths for those events without a depth but over-stacked the number of events in the first bin, producing distributions that were unsuitable for distance analysis whereas using the first quartile did not (Supplementary Fig. S3).

#### Detection function, effective strip half width (ESW), and abundance estimation

**Figure 8:**
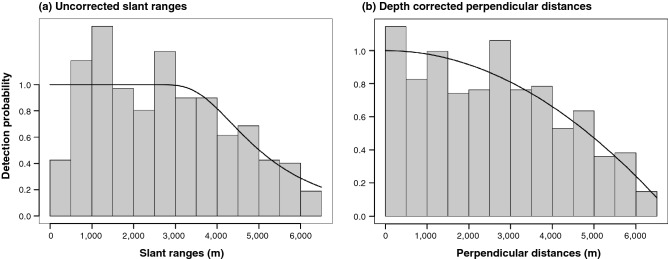
Fitted line transect models for acoustic detections of sperm whales (n = 431) truncated at 6500 m. The solid line represents the best fitting model. The histogram bars represent the (**a**) perpendicular slant range with a fitted hazard rate key function with no adjustment terms or (**b**) depth-corrected perpendicular horizontal distance with a fitted half normal key function with simple polynomial adjustment terms of order 2.

Detection functions were estimated using the truncated uncorrected slant ranges and depth-corrected perpendicular distances (n = 431) (Fig. 8). The distribution of the slant ranges was atypical for line transect sampling as the number of events in the first and second bins (0–500 m and 501–1000 m) were smaller than the third bin. All models fitted to these slant ranges failed the K–S goodness-of-fit (GOF) test at the 95% level but the preferred model passed the CvM test with a *P* value of 0.287. All models that used the first quartile fitted to these depth-corrected distances passed the K–S and CvM tests with large *P* values, indicating a good fit of the models to the data. The detection functions still violated the assumption that g(0) = 1 as bins exceeded the key function at *P* = 1 but visual examination of the q–q plots showed no outliers nor other causes of concern.

Based on the AIC, the best fitting detection function for uncorrected slant ranges was a hazard rate key function with no adjustment terms while for depth-corrected perpendicular distances it was a half normal key function with simple polynomial adjustment terms of order 2 (Table 4). Various model and adjustment combinations, including stratification by habitat type, fit the data (ΔAIC < 3) and produced similar abundance estimates (within ~ 200 animals).

**Table 4:** Results for the best five models fitted to slant ranges and depth-corrected perpendicular distances using R package Distance.

Distances	Key function	Adjustment term	AIC	ΔAIC	GOF K-S p	GOF CvM p	ESW (m) (SE)	*P* (CV%)	D (/km^2^) (CV%)	N (SE)
Slant range	Half normal	NULL	7515.933	6.728	*0*	*0.027*	4494 (217)	0.692 (4.8%)	0.00847 (14.5%)	2233.4 (232.9)
		Simple polynomial order of 2,4	7510.962	1.757	*0.03*	0.203	4953 (400)	0.762 (8.1%)	0.00769 (15.9%)	2026.9 (322.0)
		Cosine adjustment terms of order 2,3	7511.483	2.278	*0.01*	0.222	4988 (599)	0.767 (12.0%)	0.00763 (18.2%)	2012.3 (366.2)
		Hermite polynomial adjustment term of order 4	7510.543	1.338	*0*	0.107	4817 (402)	0.741 (8.3%)	0.00790 (16.0%)	2084.0 (333.9)
	**Hazard rate**	NULL*	**7509.205**	**0**	***0.02***	**0.287**	**5099 (182)**	**0.784 (3.6%)**	**0.00747 (14.1%)**	**1968.6 (278.2)**
Perpendicular distance	Half normal	Simple polynomial order of 2*	**7503.051**	**0**	**0.47**	**0.358**	**4566 (236)**	**0.702 (5.2%)**	**0.00833 (14.6%)**	**2198.6 (321.5)**
		Hermite polynomial adjustment term of order 4	7503.967	0.916	0.7	0.266	4476 (234)	0.688 (5.2%)	0.00851 (14.6%)	2242.7 (328.4)
	Hazard rate	NULL	7505.279	2.228	0.53	0.305	5029 (183)	0.774 (3.6%)	0.00757 (14.2%)	1995.9 (282.6)
		Simple polynomial adjustment term of order 2	7504.391	1.34	0.39	0.442	4694 (216)	0.772 (4.6%)	0.00811 (14.4%)	2138.5 (308.7)
		Hermite polynomial adjustment term of order 4	7504.370	1.32	0.53	0.441	4665 (224)	0.718 (4.8%)	0.00816 (14.5%)	2151.7 (311.9)

Goodness of fit (GOF) scores are based on the 95% confidence level, where *P* values that failed are italic. Asterisks and bold denotes the preferred model based on AIC. Estimates represent the number of animals using the study area during the study period (summer, 2016). Detections outside the truncation distance of 6500 m were excluded. *ESW* Effective strip half-width (m). *P* = probability of detection, *D* the total density estimate (N/area), *N* the total estimate of abundance, *CV%* the coefficient of variation. *SE* standard error.

The hazard rate model using uncorrected slant ranges resulted in an average probability of detection of 0.784 (CV = 3.6%) and an ESW of 5099 m (SE = 182 m). The half normal model using depth-corrected perpendicular horizontal distances produced a lower average probability of detection at 0.702 (CV = 5.2%) and an ESW of 4566 m (SE = 236 m), a decrease in the area effectively searched by 10.5%. The estimated number of foraging sperm whales in the study region was 1969 (CV = 14.1%, SE = 278) using uncorrected slant ranges and 2199 (CV = 14.6%, SE = 322) using depth-corrected perpendicular horizontal distances.

## Discussion

This is the first study to use passive acoustic line transect data to produce a depth-corrected acoustic abundance estimate for sperm whales in the western North Atlantic Ocean. The region where this study was conducted is an important seasonal habitat for sperm whales and is of interest for offshore wind energy development. To effectively manage and mitigate negative impacts on this species from ongoing and future anthropogenic activities, up to date and accurate knowledge of the local sperm whale distribution and density is needed.

Several previous studies^19,22,24^ evaluated the impact of not incorporating sperm whale depth in perpendicular distance estimates and concluded that any resulting bias was negligible. Barlow and Taylor^19^ calculated depth-corrected perpendicular distances for sperm whales in the Pacific Ocean using an assumed depth of 600 m which resulted in a 1% change in the ESW. In comparison, for the events analyzed in this study applying estimated and assumed depths resulted in a 10.5% change in the ESW. This suggests that the impact of using depth-corrected perpendicular distances on the output of a distance analysis could vary depending on the accuracy of the depth estimates, the dataset, and/or the population surveyed.

The acoustic abundance estimates based on slant ranges or depth-corrected perpendicular distances cannot be directly compared to those of the concurrent visual survey^5^ as the acoustic survey only sampled foraging individuals, and therefore did not count whales that were silent or socializing^19,26^. A previous tagging study^6^ in the western North Atlantic found that sperm whales spent less than 30% of their time in a non-foraging state. To account for animals missed by the acoustic survey, one approach would be to calculate a correction factor for the proportion of silent or non-foraging animals. Another approach would be to integrate visual and acoustic line transect data to estimate the availability bias and improve abundance estimation^45^.

The estimates of foraging sperm whale acoustic density in this study region were 7.5 whales per 1000 km^2^ using slant ranges and 8.3 per 1000 km^2^ using depth-corrected perpendicular distances. These are high density estimates when evaluated against other regional studies^17,22,23,46^, which have estimated acoustic densities of 0.12^22^ to 4.6^23^ sperm whales per 1000 km^2^. However, comparisons must be made with caution given the differences in methodologies. This high density is consistent with known seasonal trends in sperm whale distribution in the northwestern Atlantic, which shifts north in the summer and is concentrated between Virginia and New England where the study took place^46,47^. The distribution of the events was also similar to previous surveys conducted in the area^48,49^. Sperm whales were frequently detected along the continental slope and near Georges Bank and the Northeast Channel, which may be important seasonal foraging habitats (Fig. 4).

Using uncorrected slant ranges in the distance analysis produced a detection function that was not a good fit for the data. A significant issue was the paucity of events in the first bin of the slant range distribution; however, this was expected as the slant range can be overestimated for whales relatively close to the trackline (< 1000 m) and at depth^19,22^. The difference between the measured slant range and a whale’s true horizontal perpendicular distance will increase the closer a whale is to the trackline. Thus, it is especially important to calculate depth-corrected perpendicular distances for whales detected close to the trackline as an accurate measurement of these distances is a vital component of line transect theory^18^. Using depth-corrected perpendicular distances produced a detection function that was a better fit for the data, and supported line transect assumptions. However, when the weighted mean (620 m) or third quartile (821 m) were used as an assumed depth there was a significant increase in the first bin of distances (< 500 m) which caused issues fitting a detection function. This suggests these values were overestimates of the true depth of whales that could not be 3D localized in this study area. Applying the first quartile (411 m) improved the estimation of the horizontal perpendicular distances close to the trackline, indicating it was a closer approximation of the detected whales’ true dive depth. However, there were still small amounts of overstacking in the detection function bins. Future studies could apply a distribution of depths based on sperm whale known diving behaviour to further minimize overestimation of depth.

After correcting distances for depth, the assumption that all foraging sperm whales on the trackline were detected, a g(0) of 1, seems to be valid^17,19,24,50^. Based on a maximum vessel speed of 20 km/h and an effective detection range of 4.5 km, for a sperm whale to remain undetected it would have to be silent for more than 27 min between dives. Thus, it is unlikely that a large proportion of the foraging whales were missed^26,51^.

Events were autocorrelated, thus violating the assumption that detections were independent. This is revealed by an increase in the bin at 2501–3000 m in the depth-corrected distance distributions (Fig. 8), possibly caused by detections on several occasions of multiple whales at similar ranges from the trackline or by difficulty in resolving bearings at that distance. This analysis did not attempt to account for group size because it is difficult to accurately group individuals detected by a linear towed hydrophone array as each whale could have been to the right or left of the ship^19,23^. However, when a survey is correctly designed the assumption of independence is of minor importance and thus should not impact the robustness of these estimates^18^.

The assumption that sperm whales did not move away from the trackline before detection seems to be valid. This study found that there was no significant difference in the total number of events detected nor localized in 3D when the echosounder was in an active versus passive state and concluded that a behavioural response to the ship was unlikely. Furthermore, there are very few instances when a cessation of foraging, indicated by an abrupt end to an echolocation click train, as a result of the vessel’s presence were potentially recorded. Within the truncation distance (6500 m) 28 events (6.5%) had a duration of less than 2 min. For the events that were not interrupted by pauses or interference in the acoustic recordings, it is plausible that the whales were close to the truncation distance and not always oriented toward the hydrophone array, that the vessel hull caused an acoustic shadow zone so only a portion of the nearby and shallow dive was detected, or that there was a behavioural response to the ship.

Accurate estimates of perpendicular distances are an assumption of and vitally important for distance sampling. In this analysis the majority of the distance measurements, whether uncorrected slant ranges (90%) or depth-corrected horizontal perpendicular distances (84%), had an uncertainty smaller than 30%, suggesting this assumption has been met. Error in the slant range calculated by PAMGuard’s TMA algorithm can result from whale movement, as it is assumed the detected animals are stationary, and/or unaccounted for hydrophone array movement due to currents or wave action. Many previous studies have described these issues and ways to estimate the error or improve data collection^17,52^.

For this data, uncertainty in the measurement of the slant delay, and thus depth, was a significant factor. The method for depth estimation used in this study relies on the accurate identification of the direct click and surface reflected echo for the calculation of slant delay^31^. Interference in the recordings from wave motion, the multiple pulses of off axis sperm whale clicks, and the potential for multiple reflections from the sea surface and sea floor^53^ can all confound this process. For 2% of events that were localized in 3D, maximum click depths were recorded between 3000 and 4000 m. It is unlikely that these reflect the true maximum dive depths of the detected whales and are more likely the result of false detections of the surface reflected echo. Precision may be improved if depth estimates were rejected at a specific slant delay uncertainty; however, it would have to be tested to determine any impact on the detection distribution. A manual review of the measured slant delay was included to reduce false detections and as a result 128 (29.7%) events were excluded from 3D localization. It Is possible that these whales were at shallow depths and/or close to the hydrophone^31^, when the pulses of the direct click would overlap with the peak from the surface reflected echo.

Whether sperm whales were detected over slope (78.1%, ≤ 2000 m) or abyssal (89.3%, > 2000 m) habitat average depth was mostly between 200 and 1000 m, suggesting that foraging depth did not vary substantially based on habitat type. The depth estimates were comparable to other studies of sperm whale dive behaviour^7,26,27,51^, including a study which tagged eight female and juvenile sperm whales in the same region of the western North Atlantic and recorded an average maximum depth of 985 m (SD = 124 m) and a maximum depth of 1202 m^6^.

For the majority of the 3D localizations with U shaped click depth patterns (n = 44 events), the whale was foraging in the water column at depths > 400 m. It is possible that any detected whales foraging in the epipelagic zone (< 200 m) were excluded from 3D localization due to the difficulty in detecting surface reflected echoes when whales are at shallow depths. These 3D localizations revealed that the whales were using multiple foraging strategies by diving to prey layers in the mesopelagic (200–1000 m) and bathypelagic (> 1000 m) zones. This is consistent with previous studies which have suggested that foraging strategies vary depending on sex and age^29^, and that male sperm whales exploit multi-layered foraging grounds at high latitudes^27^. Although sea state is expected to interfere with the identification of surface reflected echoes, the duration of a detection seemed to be a more important factor in whether a partial or complete dive (descent, bottom phase, and ascent) was observable in the click depths (Supplementary Fig. S4). Future work using this method could assess if factors such as group size, distance to detected whale, and/or sea state impacted the identification of U shaped dives. If paired with demographic information such as sex or size, the results from this study could be used to assess if variations in foraging strategies occur within and/ or between male and female sperm whales. Although prey distribution in this study was not assessed, future studies could compare sperm whale presence and foraging depths with the active acoustic data from shipboard echosounders.

## Conclusion

This study demonstrates how passive acoustic line transect data can be used to produce depth-corrected acoustic abundance estimates for deep diving odontocetes like sperm whales or beaked whales, as well as answer population level questions about foraging ecology. This method of surveying a large sample of the population remotely is advantageous for assessing population-level changes. The methods and results of this study can be used to evaluate whether the distribution, acoustic abundance, or foraging strategies of sperm whales in this region change in the future and assess possible drivers such as species recovery, climate change, or anthropogenic activity including the development of offshore wind farms. An improved understanding of these dynamics is essential for current and future management and conservation planning.

## Supplementary Information

Supplementary Information.

## Data Availability

Detected sperm whale events and associated metadata are publicly available via the Northeast Fisheries Science Center’s Passive Acoustic Cetacean Map (PACM, https://apps-nefsc.fisheries.noaa.gov/pacm/#/sperm).
