# Supplementary material for: Sperm whale acoustic abundance and dive behaviour in the western North Atlantic
Source: Sci Rep. 2022 Oct 7;12:16821. doi: 10.1038/s41598-022-20868-3 (PMC9546825; doi:10.1038/s41598-022-20868-3)
Supplement: Supplementary file 1 — Supplementary Information. [file 41598_2022_20868_MOESM1_ESM.docx]

Sperm whale acoustic abundance and dive behaviour in the western North Atlantic

Annabel Westell^1*^, Taiki Sakai^2,3^, Robert Valtierra^4^, Sofie Van Parijs^5^, Danielle Cholewiak^5^, Annamaria DeAngelis^5^

^1^under contract to the Northeast Fisheries Science Center, National Marine Fisheries Service, National Oceanic and Atmospheric Administration, 166 Water Street, Woods Hole, MA 02543, USA

^2^ Environmental Assessment Services, LLC, 350 Hills St., Suite 112, Richland, WA 99354

^3^ under contract to the Southwest Fisheries Science Center, National Marine Fisheries Service, National Oceanic and Atmospheric Administration, 8901 La Jolla Shores Drive, La Jolla, CA 92037, USA

^4^Marine Acoustics Inc., 2 Corporate Pl #105, Middletown, RI 02842

^5^ Northeast Fisheries Science Center, National Marine Fisheries Service, National Oceanic and Atmospheric Administration, 166 Water Street, Woods Hole, MA 02543, USA

*Corresponding author: [annabel.westell@noaa.gov](mailto:annabel.westell@noaa.gov)

Supplementary Information

**Table S1.** Criteria used to categorize events with accepted 3D localizations (n = 265) and results including number of events per category, summary of event durations, and percent (%) time recorded in 400 m depth bins or at depths greater than 1600 m.

| **Pattern** | **Description** | **Number of events** | **Event duration,**  **min-max (mean) (min)** | **Percent (%) time spent in depth bin** | | | | |
| --- | --- | --- | --- | --- | --- | --- | --- | --- |
|  |  |  |  | **0 – 400** | **400 – 800** | **800 - 1200** | **1200 - 1600** | **>1600** |
| U shaped and shallow | Visible decrease and then increase in click depth over time; maximum click depth <800 m | 10 | 9.0-20.1 (15.0) | 53.9 | 46.1 | 0 | 0 | 0 |
| U shaped and medium depth | Visible decrease and then increase in click depth over time; maximum click depth 800 – 1600 m | 25 | 10.0-27.4 (17.9) | 20.1 | 37.2 | 33.3 | 9.4 | 0 |
| U shaped and deep | Visible decrease and then increase in click depth over time; maximum click depth >1600 m | 9 | 12.1-41.9 (24.6) | 23.0 | 18.0 | 20.6 | 15.7 | 22.6 |
| Flat and shallow | Little or no visible slope in the click depths over time; maximum click depth <800 m | 9 | 7.3-25.4 (16.8) | 98.1 | 1.9 | 0 | 0 | 0 |
| Ascending | Click depths decrease over event time | 31 | 5.3-42.5 (12.4) | 31.3 | 36.8 | 26.1 | 4.6 | 1.3 |
| Descending | Click depths increase over event time | 70 | 2.6-40.0 (11.5) | 29.8 | 34.8 | 23.8 | 6.8 | 4.8 |
| No pattern/ uncertain | No clear pattern due to intermittent detections or interference | 111 | 2.1-29.3 (9.6) | NA | | | | |


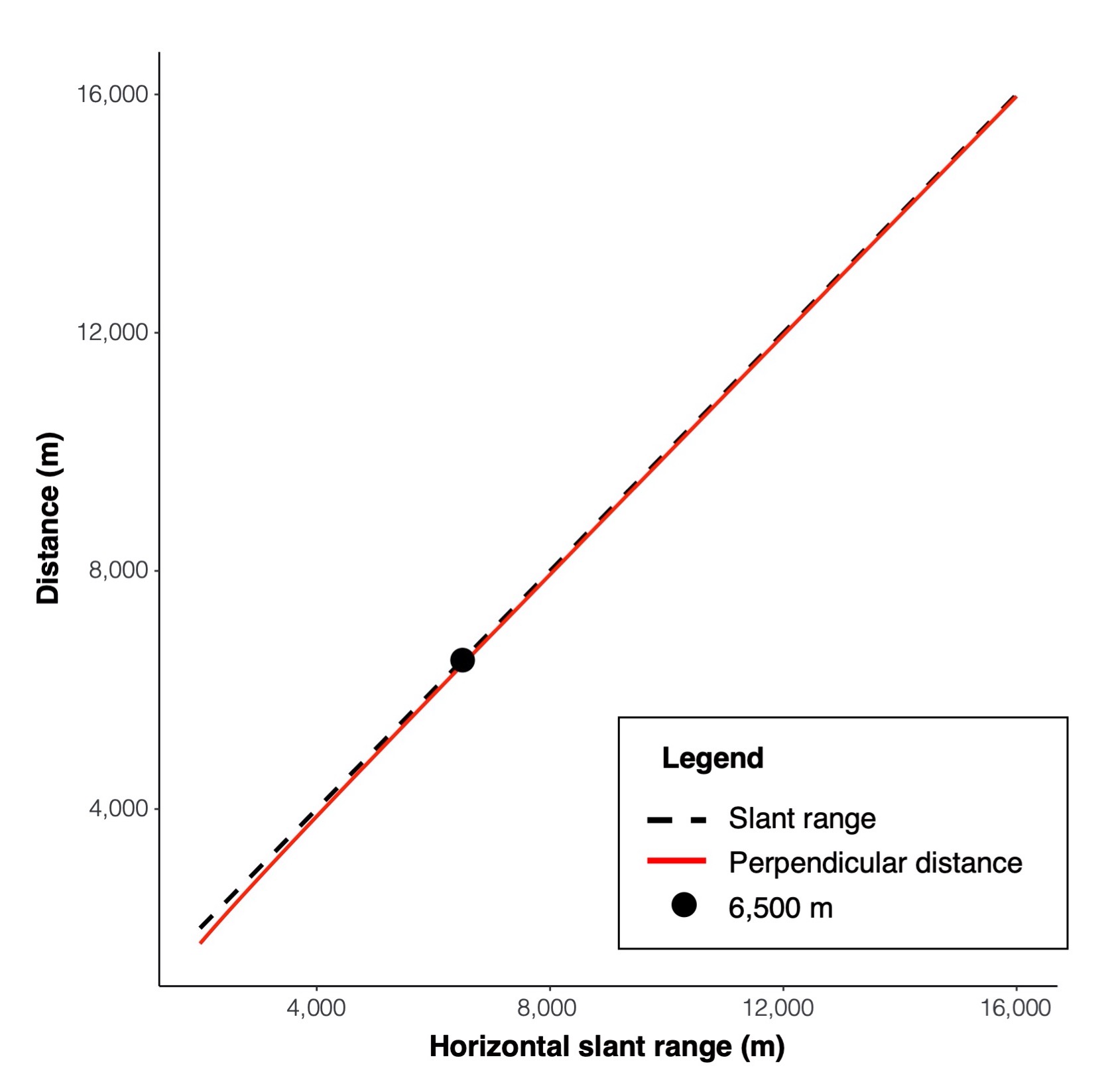


**Figure S1.** This simple simulation was used to select the truncation distance of 6500 m. An assumed constant depth of 985 m (Watwood et al. 2006) and a variable horizontal slant range between 2000 - 16000 m were used in the Pythagorean Theorem to calculate the corresponding depth corrected perpendicular distance. At a distance of approximately 6500 m, the depth of a whale is negligible in the calculation of the depth corrected perpendicular distance. Based on this, the dive depth of events with a slant range <6500 m were estimated.
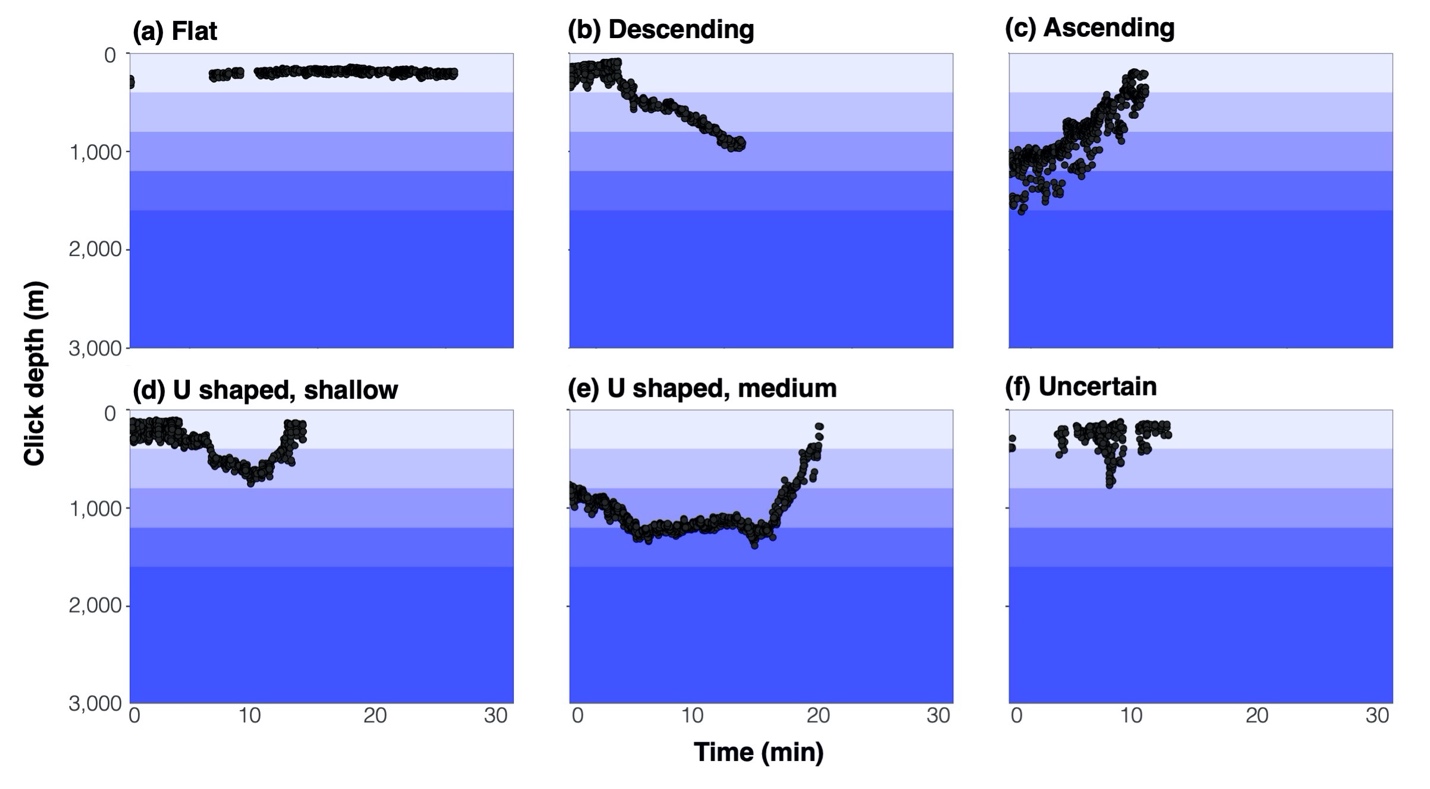
**Figure S2.** Examples of clicks depths (m) over time (min) for events categorized as (a) flat and shallow, (b) descending, (c) ascending, (d) U shaped and shallow, (e) U shaped and medium, and (f) uncertain/ no pattern.


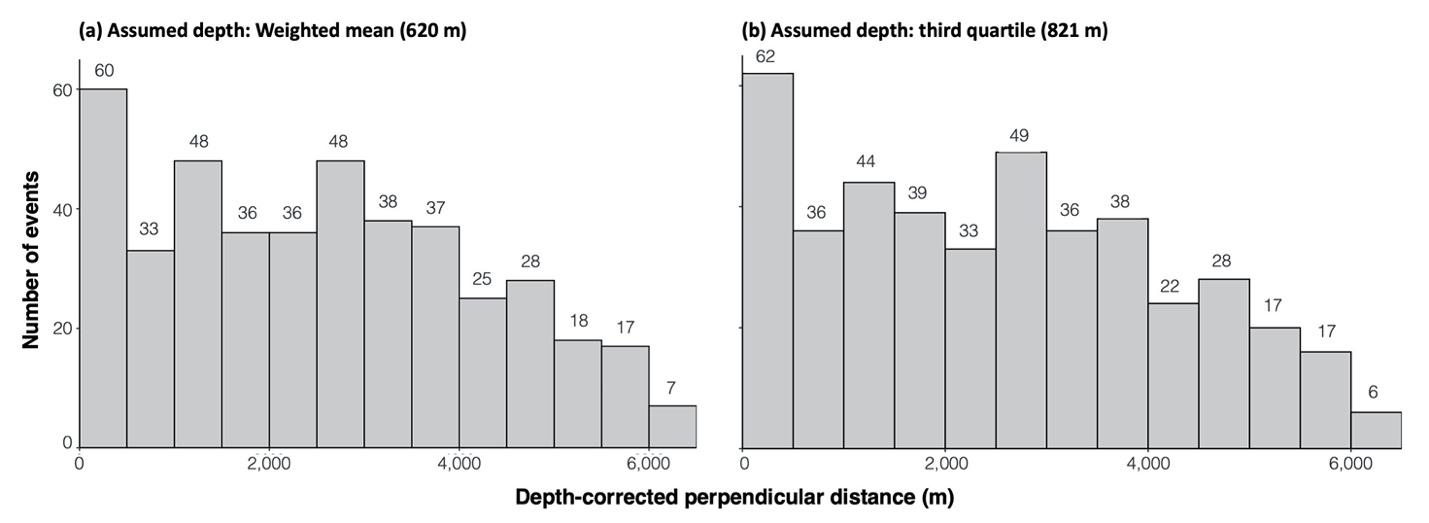


**Figure S3.** The histogram bars represent the depth corrected perpendicular horizontal distance of sperm whale detections (n = 431) calculated using the (a) weighted mean depth (620 m) or (b) the third quartile (821 m) of the average depths as an assumed depth when individual depth estimation was not possible. Applying the weighted mean depth resulted in 38 perpendicular distances coerced to zero. Applying the third quartile resulted in 45 perpendicular distances coerced to zero. The resulting distributions were not suitable for distance analysis.


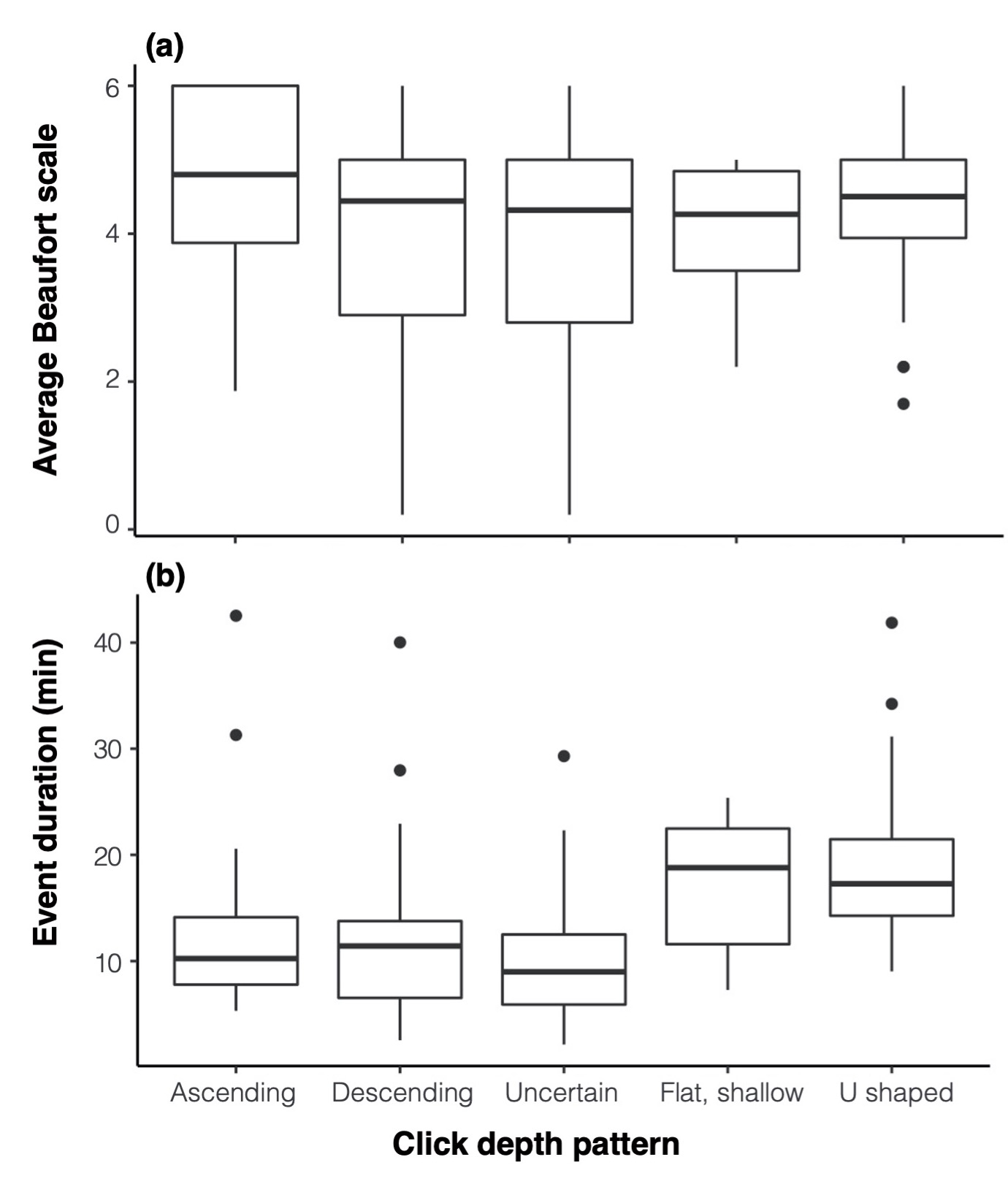


**Figure S4.** Boxplots of (a) the average Beaufort recording per event and (b) event duration (min) for events categorized based on click depth patterns as ascending (n = 31), descending (n = 70), uncertain/ no pattern (n = 111), flat and shallow (n = 9), or U shaped (n = 44).
